# Fragmentation Resilience Energy Mass Spectrometry (FREMS): Methods Validation and Compound Differentiation

**DOI:** 10.3390/molecules31020370

**Published:** 2026-01-20

**Authors:** Alexander Yevdokimov, Kevin Colizza, James L. Smith, Jimmie C. Oxley

**Affiliations:** 1 Department of Chemistry, University of Rhode Island, Kingston, RI 02881, USA; 2 GlaxoSmithKline, Collegeville, PA 19460, USA

**Keywords:** energy resolved mass spectrometry (ERMS), mass spectrometry (MS), linear ion trap (LT), Orbitrap, Fragmentation Resiliency (FR), Survival Yield (SY), structural elucidation, compound discrimination, energy calibration, cross-intersect, MS/MS, thermometer ion, glutathione, 2D-MS, 3D-MS, FREMS

## Abstract

Fragmentation Resilience Energy Mass Spectrometry (FREMS) builds on the field of energy-resolved mass spectrometry and previously used methods, e.g., Survival Yield. It exploits breakdown energies at near “continuous” ramp (0.2% NCE increments) to offer higher resolution and a reliable method for compound differentiation, contaminant identification and structural elucidation. Implementation of FREMS involves acquiring ion breakdown/formation curves as collision energy is incrementally increased. These curves themselves can be analyzed by several means to give a single metric—Fragmentation Resilience (FR_50_). This value has been shown to be experimentally interchangeable with the modified-Survival Yield (m-SY_50_) and the Cross-Intersect (C-I). A full panel of testing on an LTQ-Orbitrap revealed that breakdown energies depend only on three controllable parameters—number of ions inside the ion trap, Maximum Inject time and Activation Time. A fairly linear relationship (R^2^ > 0.95) with proposed FR_50_, m-SY_50_ and C-I metrics provides reliable adjustment mechanisms for these variables via calibrations. Consequently, this technique can be applied to ions produced by any atmospheric pressure ionization processes and treated as exclusively in vacuo experiments. Applications of FREMS to 4-chlorobenzylpyridinium ion revealed that under collisional activated dissociation (CAD) conditions, the rate of decomposition of precursor ion is equivalent to the rate of formation of its fragments, i.e., normalized breakdown and formation curves intersect at inflection points.

## 1. Introduction

Ultra-high sensitivity mass spectrometers (MS) generate superb multi-stage (MS^n^) spectra at low analyte concentrations, which can be used for structural elucidation. However, *m*/*z* fragments whose origins are not related to the isolated precursors can result in misinterpretation of molecular structures. A small subfield, termed energy-resolved mass spectrometry (ERMS), evolved alongside tandem techniques and was initially used to study “kinetic shift” effects—specifically, to measure discrepancies between theoretical and experimental energies due to the finite time required for fragmentation to occur [1,2,3,4,5,6,7,8,9,10]. As it became evident that bond dissociation energies of more complex molecules could also be probed [11,12,13], ERMS grew into a powerful methodology for quantifying the internal energy distributions produced by different ionization sources [14,15,16,17,18,19,20,21].

The survival yield (SY) method was subsequently developed as a rapid way to analyze and quantify these distributions [14]. SY is defined as the fraction of precursor ion remaining relative to the sum of the intensities of the precursor and all generated fragments (Equation (1)) [19]. In SY experiments, a well-characterized set of ionic compounds from the benzyl-substituted benzylpyridinium salt family, commonly referred to as “thermometer ions,” was selected for internal MS calibration because these species yield straightforward fragmentation patterns [20,21,22,23,24]. As energy distributions were observed to vary among compounds, SY evolved into a technique capable of differentiating closely related species, such as structural isomers [25,26,27,28,29], a distinction often unattainable by MS^n^ alone. A natural extension of this compound differentiation was the pursuit of structural elucidation using ERMS [30,31,32,33,34,35].

The SY technique relies upon the presence of both precursor and fragment ions. This precludes compounds that produced no detectable fragment(s) in the operational range of the instrument. For certain molecules, we were attempting assignment of false fragments to the precursor structures. To that end, we developed a modified SY method, which we termed Fragmentation Resilience Energy Mass Spectrometry (FREMS), with more focus on the precursor ion. Furthermore, the FREMS method offers a systematic means of separating fragment ions originating from the precursor from those arising from artifacts or impurities produced during fragmentation. This separation yields more accurate mechanistic decomposition pathways and enables improved structural elucidation.

## 2. Results and Discussion

### 2.1. FREMS—Fragment Validation and Spectral Data Cleanup

**Figure 1:**
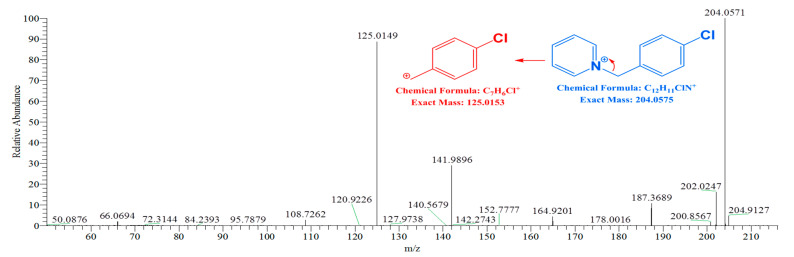
First stage MS^2^ experiments for [M]^+^ 4-ClBP; spectra are averaged over 265 scan events—NCE 0–50% range.

The addition of an energy dimension provides orthogonality to conventional MS and chromatographic approaches. The utility of FREMS for structural elucidation is demonstrated using 4-ClBP (*m*/*z* 204.0571). Figure 1 presents a first-stage (MS^2^) spectrum. Isolation and fragmentation of 4-ClBP (*m*/*z* 204) were performed in the linear ion trap, with subsequent accurate mass detection of primary “fragments” in the Orbitrap, yielding signals at *m*/*z* 202.0247, *m*/*z* 187.3689, *m*/*z* 164.9201, *m*/*z* 141.9896, *m*/*z* 125.0149. However, even high-resolution/accurate mass spectral data cannot preclude structural misassignment. Because the thermometer ions have been well characterized, it was known *a priori* that *m*/*z* 125.0149 represented the only true fragment. For unknown compounds, however, any high-intensity ion in MS^n^ experiments can mislead or complicate structural elucidation. Such ions may arise from multiple sources, including sample or instrument contamination. Accordingly, some of the observed products likely correspond to impurities with a nominal mass of *m*/*z* 204 that are not readily separated from the ion of interest within the isolation window. Simple examination of spectral traces fails to distinguish true fragments from artifacts (i.e., ions generated *during* the fragmentation process as a result of secondary or unintended reactions) or impurities (i.e., ions present in the sample prior to fragmentation, which may include impurities from the sample matrix or residual solvent), particularly those of the same nominal *m*/*z*, thereby misdirecting structural assignments and analytical effort.

**Figure 2:**
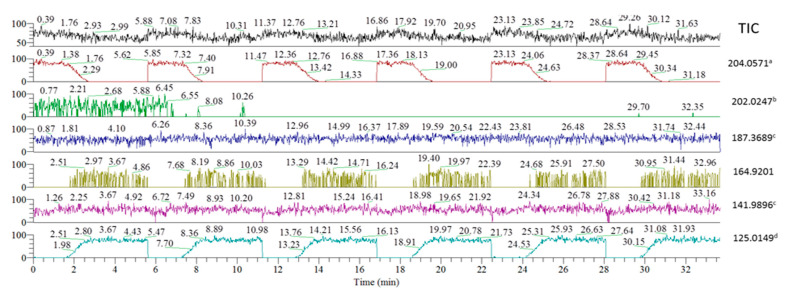
FREMS technique allows data cleanup, exemplified by analysis of 4-ClBP fragments from Figure 1, where (a) precursor; (b) contamination; (c) instrument; (d) real fragment.

The FREMS method addresses the shortcomings of current spectral tree-building approaches by incorporating proper ion selection (i.e., ions directly correlated to the precursor) into the experimental data workflow. Correlation of *m*/*z* 202.0247 to the precursor ion was highly questionable, as this would require the loss of only two hydrogen atoms. While accurate mass MS can disprove such assignments outright, nominal mass instruments may yield incorrect structural assignments. FREMS enables objective and unequivocal elimination of *m*/*z* 202.0247 as a true fragment. Examination of FREMS extracted ion chromatograms (Figure 2) reveals that this ion disappeared within fewer than two acquisition cycles. Similarly, persistent impurities present during spectral scans are readily identified using FREMS methodology because their traces do not fluctuate with energy ramps (e.g., *m*/*z* 187.3689 and *m*/*z* 141.9896). We would highlight that FREMS values need not be normalized for structural elucidation, in contrast to compound differentiation applications. The ion at *m*/*z* 164.9201 exhibited FR_50_, SY_50_, and C-I values different from those of the precursor (*m*/*z* 204.0575) and was therefore statistically eliminated as a true fragment. Precursor and fragment ions are considered correlated only if they intersect at the same FR_50_, C-I, or SY_50_ values; only *m*/*z* 125.0149 produced FREMS values consistent with those of the precursor ion (Table 1). FREMS thus provides a two-pronged solution for data cleanup and structural elucidation by focusing analytical efforts exclusively on valid precursor–fragment transitions.

### 2.2. FREMS—Differentiation of Similar Compounds

**Figure 3:**
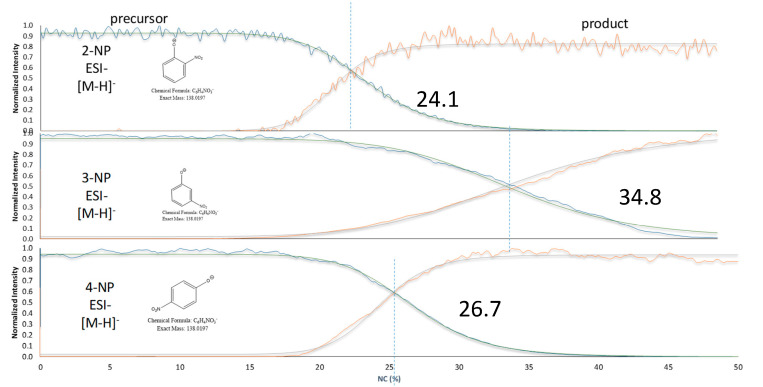
FREMS FR_50_ (shown) differentiates nitrophenols (blue precursor; red product).

**Figure 4:**
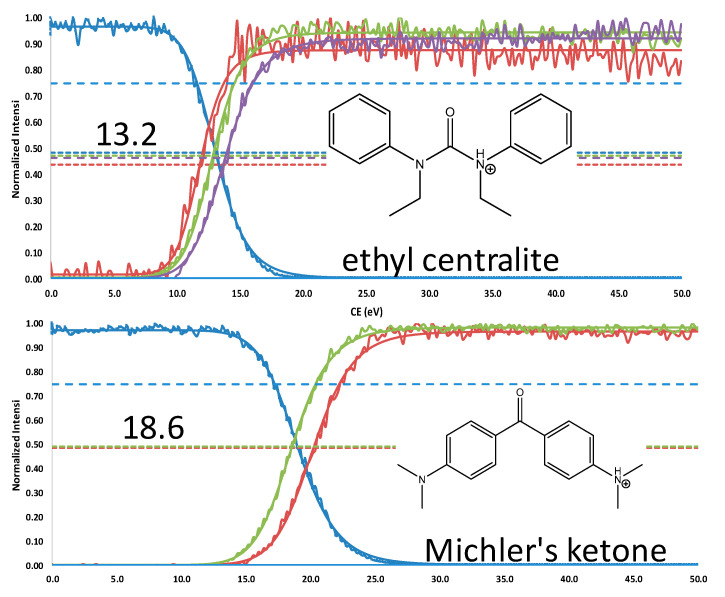
Isobaric compounds C_17_H_21_N_2_O^+^ *m*/*z* 269.1648. Nominal m/z ethyl centralite (blue) & products: red 251; green 148; purple 120. Nominal m/z Michler’s ketone product m/z: green 148; red 254.

**Figure 5:**
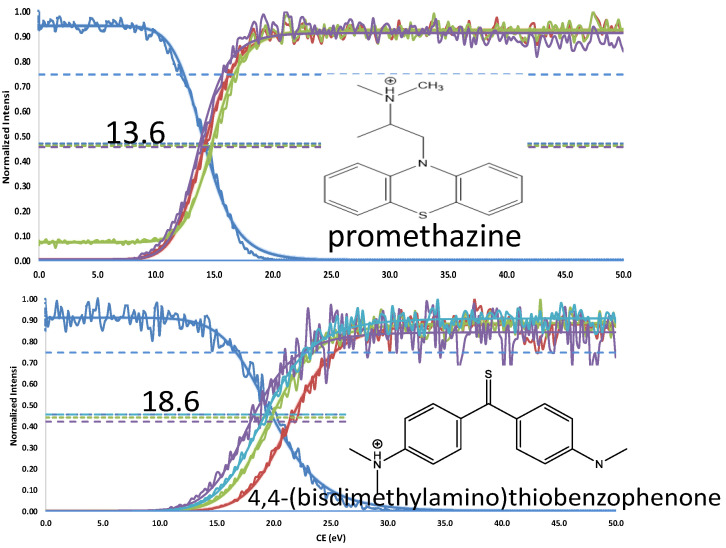
Isobaric compounds C_17_H_21_N_2_S^+^ *m*/*z* 285.1408. Nominal m/z promethazine products: red 240; green 198; purple 86. Nominal m/z 4,4-(bisdimethylamino)thiobenzophenone (blue) & products: red 270; green 252; blue 164; purple 251.

**Figure 6:**
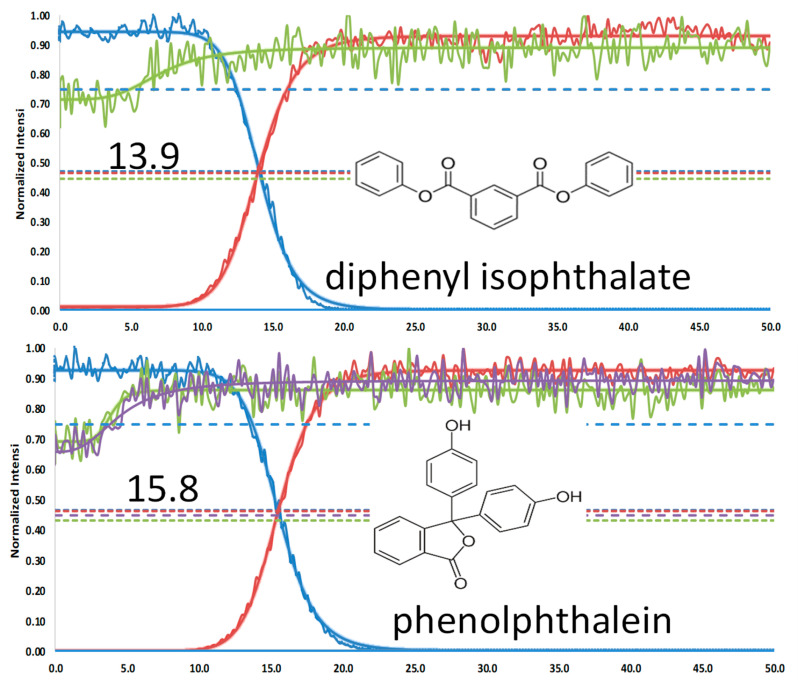
Isobaric compounds C_20_H_15_O_4_^+^ *m*/*z* 319.0964. Nominal m/z diphenyl isophthalate products: red 225; green 274.9; Nominal m/z phenolphththalein (blue) & products: red 225; green 300; purple 274.9.

The nitrophenol isomers (2-, 3-, and 4-NP) share a common precursor ion at *m*/*z* 138.0197 [M-H]^−^ and, regardless of the ionization mode (ESI^−^ or APCI^−^), produce a single fragment at *m*/*z* 108.0210 [M-NO]^●−^ during MS/MS experiments, resulting in indistinguishable spectra (Figure S2). However, by applying the FREMS framework, these compounds can be differentiated (Figure 3). This differentiation is particularly valuable for isobaric compounds, some of which yield identical principal ions. We applied FREMS to three sets of isobaric compounds to illustrate how this approach also provides structural information (see Figure 4, Figure 5 and Figure 6). This capability is especially evident in Figure 6, where diphenyl isophthalate readily loses CO_2_ with no energy input, whereas phenolphthalein loses both CO_2_ and water.

When several fragments exhibit FREMS values (FR_50_, m-SY_50_, C-I) similar to that of the dissociating precursor, decomposition routes must account for this possibility. For various reasons, fragments appearing at the first dissociation stage (MS^2^) may not truly belong to that stage. Prior to performing second-stage mass spectrometry, FREMS figures (e.g., Figure 4, Figure 5 and Figure 6) provide an initial indication that a particular ion may not belong to the first-stage decomposition. The ion at *m*/*z* 270, represented by the red trace in Figure 5, is observed at considerably higher energy than the other fragments and exemplifies this phenomenon. This feature will be discussed in detail in a follow-on paper.

### 2.3. FREMS Method Validation

#### Mass Spectrometer Parameters

**Figure 7:**
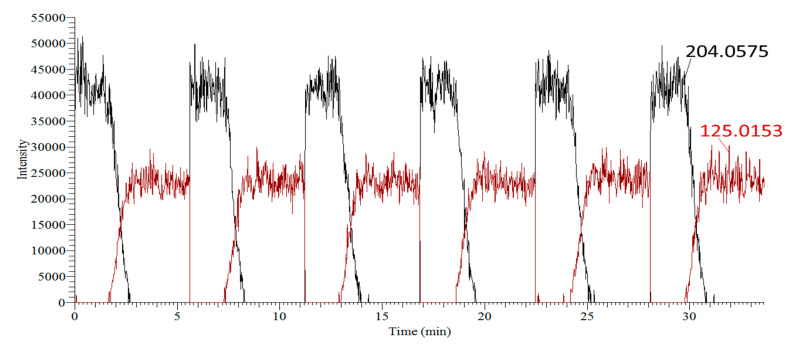
Precursor breakdown and fragment formation curves for 4-chloro-benzylpyridinium ion (4-ClBP; [M]^+^, *m*/*z* 204.0575), without normalization (n = 6 replicates).

A model compound, 4-chlorobenzylpyridinium (4-ClBP; [M]^+^, *m*/*z* 204.0575), was used to investigate all MS-controllable parameters and ascertain the extent to which this technique enables compound differentiation. The FREMS values (FR_50_, m-SY_50_, C-I) calculated from ion breakdown curves, such as that shown in Figure 7, exhibited extremely small standard deviations, particularly for the statistical m-SY and FR methods (Table S1 for *p*-values). Ideally, all three methods should produce identical results; however, to demonstrate method robustness and consistency, it was more important that each could be repeated precisely.

Certain parameters, such as collision gas pressure and temperature, could not be controlled on our instrument but have been reported to affect breakdown energies [29]. To account for uncontrollable shifts and deviations due to concentration, a daily single-point calibration was performed. Exhaustive trials were conducted applying FREMS methodology to 4-ClBP, varying every parameter within the analyst’s control. No statistically significant differences were found between breakdown values of precursor ions across all FREMS methods when the same intensity (concentration) levels were considered (Table S1 and Figure S4 for *p*-values). Thus, all three methods can be used interchangeably. More importantly, we found that FREMS values differing by >0.3% NCE are unique and allow differentiation of closely related compounds.

**Figure 8:**
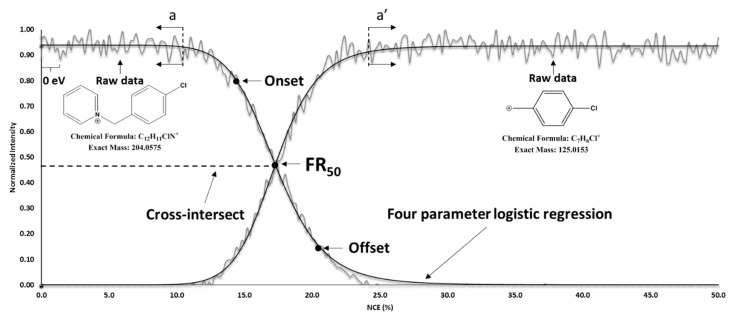
FREMS applied to 4-ClBP ion and fragment show identical FR_50_ & C-I values. Onset and Offset may be truncated at a & a’.

Furthermore, because precursor decomposition must be mirrored by product appearance, their FREMS values must be equivalent. The statistical difference between FR_50_ values of the precursor (*m*/*z* 204.0575) and fragment (*m*/*z* 125.0153) ions (i.e., 0.2–0.4% difference) was attributed to noise levels. Truncation of the breakdown curve at the highest asymptote regions (Figure 8, a and a′) would improve analysis by minimizing value spread due to S/N differences.

To fully test the applicability of FREMS methods, an inter-instrument comparison is typically required. Although no access to an identical instrument was available, a fortuitous, albeit costly, event occurred approximately one month into data collection when the instrument catastrophically failed. A plasma flash necessitated complete replacement of the ion trap, C-trap, and ion optics, resulting in a virtually new instrument. Demonstrating the robustness of FREMS methods, no significant difference (*p*-value > 0.05 for all observations) was found between two data sets collected under standard conditions 360 days apart, before and after repair (Table 2, day 5 compared to day 365).

### 2.4. Analyte Concentration

As shown in Table 2, FR_50_, m-SY_50_, and C-I values shifted to higher breakdown energies as analyte concentration increased. Indeed, ion populations greater than 10^5^ have been shown to contribute to space charge effects in ion traps, adversely affecting trapping capacity, mass discrimination, resolution, and fragmentation efficiency [36,37,38]. Consistent with these reports, FR_50_, SY_50_, and C-I energies were stable up to approximately 10^5^ intensity and then shifted significantly (*p*-values < 0.05, Figure S3). We hypothesize that greater ion populations (i.e., higher concentrations) create denser ion clouds that shield the main ion core from fragmentation at the same energies required for more dispersed clouds.

Fortunately, FR_50_, SY_50_, and C-I demonstrated a linear dependence on intensity over the entire tested range (four orders of magnitude), with R^2^ > 0.98. Other metrics, such as onset and offset values for FREMS, also exhibited a strong linear relationship (R^2^ > 0.94, Figure S5). Therefore, the small breakdown energy variation below 10^5^ intensity was likely attributable to minor ion population fluctuations within the ion trap. To determine the appropriate range for a specific sample, an energy calibration should be performed across the desired intensity range.

Because atmospheric pressure ionization (API) techniques occur in an open environment, achieving high levels of consistency and reproducibility is challenging. These limitations hinder the creation of unified spectral libraries analogous to the NIST Mass Spectral Library for compound identification via GC-MS. FREMS is purely MS-based, performed under vacuum, and decoupled from the initial ionization process for a specific precursor ion (note: API in addition can produce different charge states and adduct formation that can also be analyzed by FREMS); thus, as long as ion trap MS parameters remain constant, trapping resets the ion energy to the same state at the initiation of each MS^2^ experiment. This ensures reproducible results and consistent fragmentation patterns.

To ascertain the effects of front-end conditions on breakdown energies, all accessible conditions were tested (Table S2, Figure S6). All experiments demonstrated that front-end variables affect only intensities and exhibit a linear relationship to FR_50_, SY_50_, and C-I values. Using an energy calibration curve (R^2^ = 0.98–0.99), intensity-adjusted parameters were no longer statistically significant. No statistical significance (α = 0.05) was observed between FR, m-SY, and C-I methods (*p*-value > 0.05) for 27 of 29 pairs. Outliers resulted from low intensity and decreased S/N under high sweep gas flow and high in-source CID conditions, causing poor curve fitting.

Settings controlling the number of ions entering the ion trap (IT) or Orbitrap (FT) had an effect roughly proportional to analyte concentration, provided the ion count remained between 10^3^ and 10^7^. With the exception of FT-MS^n^, which occurs in the Orbitrap, none of the IT or FT population parameters affected FR_50_, SY_50_, and C-I (*p*-value > 0.05, n = 38); each was tested at extreme levels (Tables S3 and S4). To avoid artificial energy shifts, operation within an ion range of 5 × 10^4^ to 10^7^ is recommended.

Time parameters, such as Maximum Inject Time (MIT), which controls ion batch accumulation duration before detection, and Activation Time (AT), which sets the RF application duration, affected FR_50_, SY_50_, and C-I values in FT-MS^n^ but not in other modes (Figure S7, Tables S5–S7). MIT was fixed at 10 ms, and AT was set to 30 ms. Wideband Activation, a parameter providing RF voltage across a wide range of excitation frequencies, and Activation Q, which allows adjustment of the RF frequency used in fragmentation, were also held constant. The Microscans (μs) parameter, which determines how many scans are summed to produce a single output, was found to alter acquisition times without affecting FREMS values and was set to 1 μs. (Tables S8 and S9) The remaining controllable parameters—Mass Range, Data Type, and Resolution—had no effect on FREMS values (*p*-values > 0.05 for all, Table S10); lower resolution reduced acquisition time by decreasing transient times inside the Orbitrap.

The FREMS approach described here was developed and validated on a trap-type, accurate-mass mass spectrometer. Its application proved particularly valuable for fragment identification when using nominal-mass detectors. In linear ion trap MS/MS, primary fragments are generated in a controlled manner, greatly simplifying structural interpretation. In such instruments, once an ion of a specific *m*/*z* is trapped, its fragmentation is induced by applying a resonant RF frequency. The deposited energy—modulated by adjusting the amplitude—promotes vibration and collision with an inert gas (e.g., helium) in a process referred to as *collisionally activated dissociation* (CAD) [36,39,40]. Under these conditions, product ions generally do not fragment further unless they retain residual energy, since they do not resonate at the same frequency as the precursor. This mechanism is distinct from *collision-induced dissociation* (CID), used in instruments such as triple quadrupoles or HCD cells, where precursor and fragment ions undergo continuous, higher-energy collisions via a voltage differential [41,42,43,44].

The FREMS methodology enables the separation of precursor-derived fragment ions from artifacts and impurities generated during CAD fragmentation. This yields more accurate mechanistic decomposition pathways and enhances structural elucidation. The method was developed and validated on a trap-type mass spectrometer; its current application is therefore specific to this platform. Future work is required to evaluate its applicability to other instrument types with fundamentally different mass isolation and fragmentation principles, such as time-of-flight or triple-quadrupole mass spectrometers.

## 3. Experimental Section

Reagents. Optima^TM^ LC/MS acetonitrile, methanol, and water were purchased from Fisher Scientific (Fair Lawn, NJ, USA). The nitrophenols, diphenyl isophthalate, 4,4-(bisdimethylamino)thiobenzophenone, ethyl centralite, phenolphthalein, Michler’s ketone, and promethazine were purchased from Millipore Sigma (Burlington, MA, USA) 4-Chlorobenzyl-pyridinium salt (4-ClBP) was prepared as reported [45].

Instrumentation. Experiments were performed on Thermo Scientific (Waltham, MA, USA) LTQ Orbitrap XL^TM^ in MS/MS mode using direct infusion with Hamilton syringe (i.d. 4.61 mm). Specific *m*/*z* isolation was performed using linear ion trap and detected by Orbitrap. All controllable MS parameters were tested to verify impact on breakdown energies. Standard conditions (STM) were as follows: sheath = 15(arb); auxiliary gas = 3; sweep gas = 0; ionization voltage = 4.5 kV; FTMS^n^ Ion Population Injection= 1E5; FT Inject Time = 100 ms; Activation Time = 30 ms; Wideband Activation off; Activation Q = 0.25; acquisition time =33 min; # of FREMS breakdown curves = 6; more details can be found in Tables S1–S10. After ionization source parameters were chosen, a concentration corresponding to a moderate intensity (1-2E06) was selected for all experiments to produce good signal-to-noise.

Methods Description: Survival Yield (SY) applies a few discrete breakdown energies to the precursor ion. To better differentiate closely related compounds, we increased the energy resolution by raising the collision energy in 0.2% steps, as follows. First, an ion of interest was trapped inside the LTQ with no external energy added (0% normalized collision energy, NCE). To establish the baseline, 15 scans were collected at 0% NCE. This allowed averaging of spectra for assessing background thus providing a better algorithm fitting for ions that had extremely low onset breakdown. Following initial baseline collection, an energy ramp was employed. To ensure continuous data collection, the number of scans was maximized within one segment on a Thermo LTQ-Orbitrap^TM^ MS (Thermo Scientific, Waltham, MA, USA). In that segment, 15 scans were dedicated to background collection (0% NCE), with 250 scans going from 0% to 50% NCE in 0.2% increments, for a total of 265 scheduled scans in a single run. This was enough energy to completely fragment all tested compounds (Figure 7). Replicates were collected to perform statistical analysis and improve signal-to-noise ratios. Breakdown curves and formation curves are shown in Figure 7 with six replicates. Although breakdown curves have been used in ERMS and SY applications, overlaying these curves using maximum intensity for all ions visibly obscures correlation [14,16,17,19,32,33] since the intensity of the product ion is usually significantly lower than the precursor (Figure S1).

Normalizing individual traces to the same scale (e.g., 0–1, or 0–100, Figure 7) provides direct observation of trends (Figure 8). To normalize, all data points of the precursor and of each fragment were individually scaled to their highest intensity (for the precursor ion the first 15 points were collected at 0% and averaged). Data was smoothed by averaging the same energy levels from replicate runs, e.g., 20th point from each curve in Figure 7 (n = 6) to produce a single 20th point in Figure 8. This method suffers less skewness than boxcar averaging.

As illustrated in Figure 8, as NCE in the ion trap is increased incrementally from 0% to 50%, in some instance, the ions in resonance gain enough energy to initiate breakdown via collisions with the inert gas (e.g., He). The characteristic NCE value at which this occurs is labeled the Onset point (Figure 8). This point reveals the susceptibility of a given ion to begin dissociating, a property we term fragmentation resilience. When this resilience is probed continuously across a range of collision energies, the resulting methodology is referred to as Fragmentation Resilience Mass Spectrometry (FREMS).

The Onset point, together with the complementary Offset point (where the fragmentation yield saturates), delineates approximately 80% of the linear region of the fragmentation curve. This region reflects both the steepness and curvature of the response, enabling clearer interpretation and more reliable cross-comparison among results—particularly for distinguishing closely related ion structures. Moreover, the Onset point is relatively straightforward to identify owing to the high signal-to-noise ratio typically observed at the onset of dissociation.

In applying energy-resolved mass spectrometry (ERMS), the breakdown curves display a sigmoidal shape, reflecting the gradual transition from intact precursor ions to complete fragmentation as the collision energy increases. A logistic model is well suited to describing this behavior because it captures the nonlinear, saturating response of fragmentation probability with respect to energy. The logistic function provides parameters with clear physical meaning: the midpoint corresponds to the energy at which 50% fragmentation occurs (i.e., fragmentation resilience at 50% or FR_50_), while the slope reflects the sharpness of the transition. The four-parameter logistic regression (Equation (2)) was used to model the ion breakdown curves, where *a* is the lowest asymptote; *b*, the Hill’s slope (instantaneous slope at inflection point); *c*, the inflection point (FR_50_); and *d*, the highest asymptote. This equation was used to calculate the FR_50_ value in the FREMS technique.

(1) $$y=d+\frac{a-d}{1+{\left(\frac{x}{c}\right)}^{b}}$$

(2) $$SY=\frac{I_{p}}{I_{p}+\sum I_{f,i}}$$

The least squares statistical method for minimizing the sum of residual errors was employed to model the data. For formation curves, all parameters were reversed, meaning onset was 10% and offset was 90%, while the sign of the slope changed. [The specificity of collisional activated dissociation (CAD) prevents fragments from being further broken down; thus, sigmoidal curves were observed]. The value of the inflection point, FR_50,_ is the most important feature for quick differentiation among compounds. Other parameters, e.g., lowest and highest asymptotes, are essentially the same for all observation, 0 and ~1, respectively. FR_50_, in the middle of the breakdown curve, combines onset and offset points into a single value that we use as a metric for differentiation among compounds.

If no fragments are detected in the range of the MS instrument, the Survival Yield (SY) method is not applicable. If ions not arising directly from the precursor are included in the analysis the SY is misleading as the values are shifted to lower apparent energies. [33] However, once only true fragments are identified, the inflection point of the SY curve is equivalent to that of the calculated FR_50._ This also is the point at which the precursor fragmentation curve should intersect the fragment formation curve (C-I) (Figure 7, Table 2).

Once the precursor ion is fully dissociated, no new fragments are generated. Consequently, even for a precursor with multiple decomposition pathways, when the collision energy reaches the point where the precursor intensity is reduced to 50% of its original value, each of its primary fragment ions should also be at half of its maximum intensity. The mid-point of the fragmentation curve is where the slopes of the precursor and fragment intensities intersect, and this should coincide with the FR_50_. This intersection can be determined by a graphical (non-statistical) approach we term cross-intersection (C-I). For species creating multiple fragments, each related fragment must cross-intersect at the FR_50_. If they do not (if they are well above or below mid-point), then these fragments belong to a different precursor or, in some cases, a different MS stage. The modified survival yield (m-SY) technique is essentially the standard SY calculation performed after unrelated fragments have been removed. This removal is achieved by first applying the FREMS/C-I step, which identifies and excludes fragments not linked to the primary precursor, thereby refining the SY measurement and avoiding potential misinterpretations. Therefore, the m-SY technique may generate the same m-SY_50_ as the FR_50_ from the FR technique if the fragments being evaluated are correctly associated. In this paper, the FREMS technique will include the calculated or observed values for the FR_50_, m-SY_50_ and C-I.

## 4. Conclusions

Fragmentation Resilience Energy Mass Spectrometry (FREMS) functions by filtering or trapping ions of interest and subjecting them to a controlled, ramped energy deposition. Analysis of the resulting FREMS-extracted ion chromatograms enables the clean separation of true product ions from artifacts and impurities, accelerating data interpretation. This method effectively differentiates closely related species, including regioisomers and isobaric compounds, by leveraging an energy-based approach that provides structural insights traditionally gained only through subsequent MS/MS experiments.

Robustness was established using 4-chlorobenzylpyridinium under CAD conditions on an ion trap–orbitrap platform, confirming that the precursor decomposition rate directly correlates with true fragment formation. This allows FREMS to filter out impurity-related ions, focusing the analysis on structurally relevant fragments and improving elucidation accuracy. Conceptually, FREMS builds upon and extends the framework of the survival yield (SY) method by more fully exploiting the energy dimension in mass spectrometry.

Performance evaluation showed that, in most cases, the derived parameters (FR_50_, C-I, and m-SY) produced statistically equivalent results (*p* > 0.05 for 220 out of 236 pairwise observations). Minor deviations observed between the C-I and m-SY methods were attributable to low signal-to-noise data rather than methodological differences. While the ionization process influences absolute ion counts, it demonstrated an exceptional linear dependence across four orders of magnitude (R^2^ > 0.99), enabling reliable calibration and quantification. Critically, once signal intensities of the trapped precursor ion were normalized, the ionization source no longer exerted a statistically significant impact on FREMS results, effectively reducing the influence of ionization variability and improving repeatability.

The long-term reproducibility of the method was validated over a 360-day period, during which ion fragmentation optics were replaced, with no statistically significant variation observed (*p* > 0.05 for all comparisons). This demonstrates that consistent product ion formation can be achieved under standardized experimental conditions. Only the *Maximum Injection Time* and *Activation Time* parameters were found to directly influence absolute FREMS values; however, these also exhibited a highly linear relationship (R^2^ > 0.95), meaning their effects can be predicted and accounted for.

### Broader Implications and Future Directions

FREMS provides a reproducible, energy-resolved framework that reduces the influence of ionization-related variability, establishing a pathway toward the development of standardized, energy-dependent spectral libraries. Such libraries would substantially streamline compound identification in complex mixtures, with direct applications in fields such as metabolomics, environmental analysis, and forensic science. The method’s reproducible performance under controlled experimental conditions further supports its potential for integration into high-throughput, multi-omics, and non-targeted screening workflows.

Future research will focus on validating FREMS across a broader range of compound classes using ion trap–orbitrap systems, while also exploring its adaptability to other mass spectrometry configurations. Work is currently in progress to adapt and apply FREMS methodologies to collision-induced dissociation (CID)-based platforms, which would further extend its analytical utility.

## Figures and Tables

**Table 1:** NCE% values all combined curves 4-ClBP, *m*/*z* 204.0571 and fragment, *m*/*z* 125.0149 (n = 6 replicates, standard deviation).

			FR_50_	
NI	C-I	m-SY_50_	*m*/*z* 204.0571	*m*/*z* 125.0149
6.66 × 10^5^	17.7 ± 0.2 (17.6)	17.6 ± 0.1 (17.6)	17.6 ± 0.1 (17.6)	17.5 ± 0.1 (17.5)

**Table 2:** FREMS dependence on concentration (as intensity) of 4-ClBP’s, using Thermo Scientific LTQ-Orbitrap XL^TM^, Thermo Scientific, Shirley, NY, USA.

		FREMS			
Day	NI (counts)	C-I (NCE %)	*m*-SY_50_ (NCE %)	FR_50_ (NCE %) *m*/*z* 204.0575	FR_50_ (NCE %) *m*/*z* 125.0153
1	6.33 × 10^4^	17.8 ± 0.3 ^a^ (17.6)	17.8 ± 0.1 (17.8)	17.8 ± 0.1 (17.8)	17.7 ± 0.1 (17.6)
2	7.13 × 10^4^	17.7 ± 0.2 (17.6)	17.7 ± 0.1 (17.7)	17.7 ± 0.1 (17.7)	17.5 ± 0.1 (17.5)
2	1.94 × 10^5^	17.9 ± 0.4 (17.6)	17.8 ± 0.3 (17.7)	17.8 ± 0.3 (17.8)	17.6 ± 0.2 (17.4)
2	4.07 × 10^5^	17.8 ± 0.2 (17.6)	17.7 ± 0.2 (17.7)	17.8 ± 0.1 (17.8)	17.5 ± 0.1 (17.5)
2	5.05 × 10^5^	17.8 ± 0.3 (17.6)	17.6 ± 0.3 (17.7)	17.9 ± 0.1 (17.9)	17.5 ± 0.1 (17.5)
5	5.70 × 10^5^	18.1 ± 0.2 (17.8)	17.7 ± 0.2 (17.8)	18.0 ± 0.1 (18.0)	17.5 ± 0.2 (17.6)
5	6.98 × 10^5^	18.0 ± 0.3 (18.2)	17.8 ± 0.1 (17.8)	17.9 ± 0.1 (18.0)	17.6 ± 0.1 (17.6)
5	7.83 × 10^5^	17.8 ± 0.3 (18.2)	17.8 ± 0.1 (17.8)	17.9 ± 0.1 (17.9)	17.6 ± 0.0 (17.6)
5	8.84 × 10^5^	18.0 ± 0.3 (18.2)	17.8 ± 0.1 (17.9)	18.0 ± 0.1 (18.0)	17.6 ± 0.1 (17.6)
5	9.41 × 10^5^	18.2 ± 0.0 (18.2)	18.0 ± 0.1 (17.9)	18.0 ± 0.0 (18.0)	17.7 ± 0.1 (17.7)
5	1.09 × 10^6^	18.2 ± 0.0 (18.2)	18.0 ± 0.1 (18.0)	18.1 ± 0.1 (18.1)	17.8 ± 0.1 (17.8)
5	1.20 × 10^6^	18.2 ± 0.0 (18.2)	18.0 ± 0.1 (18.0)	18.2 ± 0.1 (18.2)	17.8 ± 0.0 (17.8)
5	1.33 × 10^6^	18.2 ± 0.0 (18.2)	18.1 ± 0.0 (18.0)	18.2 ± 0.1 (18.2)	17.9 ± 0.1 (17.9)
5	1.66 × 10^6^	18.4 ± 0.2 (18.2)	18.3 ± 0.1 (18.2)	18.4 ± 0.1 (18.4)	18.0 ± 0.0 (18.0)
5	1.97 × 10^6^	18.6 ± 0.0 (18.6)	18.4 ± 0.0 (18.3)	18.4 ± 0.0 (18.4)	18.1 ± 0.0 (18.1)
5	2.22 × 10^6^	18.6 ± 0.0 (18.6)	18.4 ± 0.1 (18.5)	18.5 ± 0.1 (18.5)	18.2 ± 0.0 (18.2)
6	2.14 × 10^6^	18.6 ± 0.0 (18.6)	18.4 ± 0.1 (18.4)	18.5 ± 0.1 (18.6)	18.2 ± 0.1 (18.2)
14	8.71 × 10^6^	20.4 ± 0.1 (20.4)	20.2 ± 0.0 (20.2)	20.4 ± 0.1 (20.4)	20.0 ± 0.1 (20.0)
14	1.17 × 10^7^	21.2 ± 0.2 (21.2)	21.0 ± 0.1 (21.0)	21.2 ± 0.2 (21.2)	20.8 ± 0.1 (20.7)
365 ^c^	1.96 × 10^6^	18.8 ± 0.3 (18.8)	18.6 ± 0.1 (18.5)	18.4 ± 0.1 (18.4)	18.2 ± 0.1 (18.4)
365 ^c^	2.13 × 10^6^	18.9 ± 0.2 (18.8)	18.8 ± 0.1 (18.7)	18.5 ± 0.1 (18.5)	18.4 ± 0.2 (18.4)

Day—days since first experiment; NI—normalized intensity; C-I—cross-intersect method; SY_50_—inflection of *m*-SY curve; FR_50_—inflection of FR curve; ^a^ standard deviation (n=6 replicates); ^c^ after repair.

## Data Availability

The original contributions presented in this study are included in the article/Supplementary Materials. Further inquiries can be directed to the corresponding author.

## Supplementary Materials

The following supporting information can be downloaded at: https://www.mdpi.com/article/10.3390/molecules31020370/s1, Figure S1. Overlay of TNT precursor and its fragment ions without normalization factor (n = 6 replicates); Figure S2. MS^2^ spectra for precursor ions of mono-nitrotoluene; Figure S3. Measure of significance (*p*-values) for FR_50_ (Table 2) using one-factor ANOVA; Figure S4. 4-ClBP’s FR_50_, SY_50_ and C-I dependence on concentration (expressed as intensity) from Table 2; Figure S5. Dependence of Onset, FR_50_ and Offset parameters on concentration (expressed as intensity) for Fragmentation Resilience method for 4-CIBP; Figure S6. FR_50_ dependence on (a) gas flows and in-source CID; (b) ion optics and ionization source housing; Figure S7. FR_50_ relative to (a) Maximum Inject Time (b) Activation Time, in FT-MS^n^; Table S1. Statistical significance (*p*-values at α = 0.05) between breakdown energies of FR_50_, SY_50_ & C-I methods (Table 2) at different intensities; Table S2. Dependence of FREMS values on source parameters; Table S3. Effects of Orbitrap’s Automatic Gain Control Ion Population parameters; Table S4. Effects of linear ion trap’s Automatic Gain Control Ion Population parameters; Table S5. Effects of Inject Times (ms) in Orbitrap analyzer on FREMS values; Table S6. Effects of Inject Times (ms) in IT analyzer parameters; Table S7. Dependence of FREMS values on Activation Time (ms); Table S8. Effects of number of Microscans in FT analyzer; Table S9. Effects of number of Microscans in IT analyzer; Table S10. Effects of Scan Range, Resolution and Data Acquisition Type.
