# Supplementary material for: Fragmentation Resilience Energy Mass Spectrometry (FREMS): Methods Validation and Compound Differentiation
Source: Molecules. 2026 Jan 20;31(2):370. doi: 10.3390/molecules31020370 (PMC12844248; doi:10.3390/molecules31020370)
Supplement: Supplementary file 1 [file molecules-31-00370-s001.zip › molecules-4004014-supplementary.pdf]

Supplemental Information:

Fragmentation Resilience Energy Mass Spectrometry (FREMS): Methods Validation and Compound Differentiation

Alexander Yevdokimov [1], Kevin Colizza [2], James L. Smith [1] and Jimmie C. Oxley [1,*]

[1] Department of Chemistry, University of Rhode Island, Kingston, RI 02881, USA; ayeudakimau@gmail.com (A.Y.); jlsmith1@uri.edu (J.L.S.)
[2] GlaxoSmithKline, Collegeville, PA 19460, USA; kcolizza@gmail.com
* Correspondence: joxley@chm.uri.edu

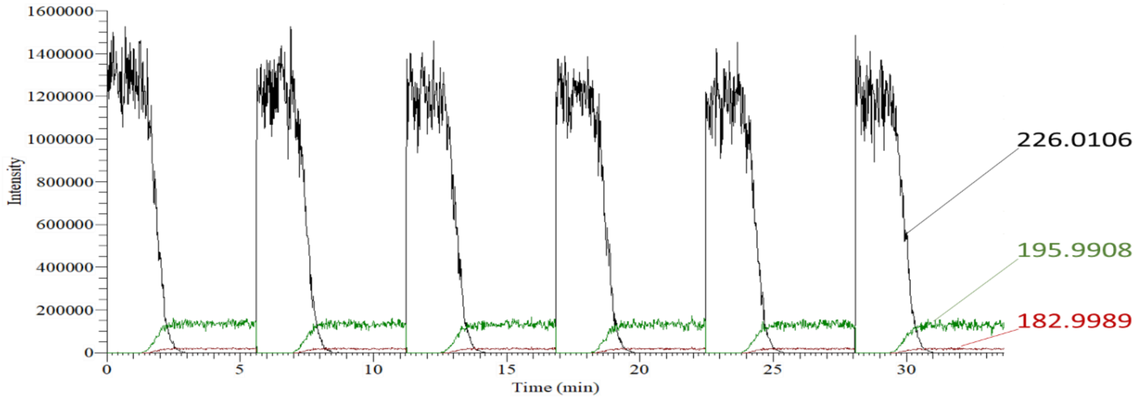

Figure S1. Overlay of TNT precursor and its fragment ions without normalization factor (n = 6 replicates). Intensities differences between TNT's ion, m/z 226.0106, and its fragment, m/z 182.9989, prevent any meaningful visual examination. As discussed, product fragment intensities were normalized to the precursor ion.

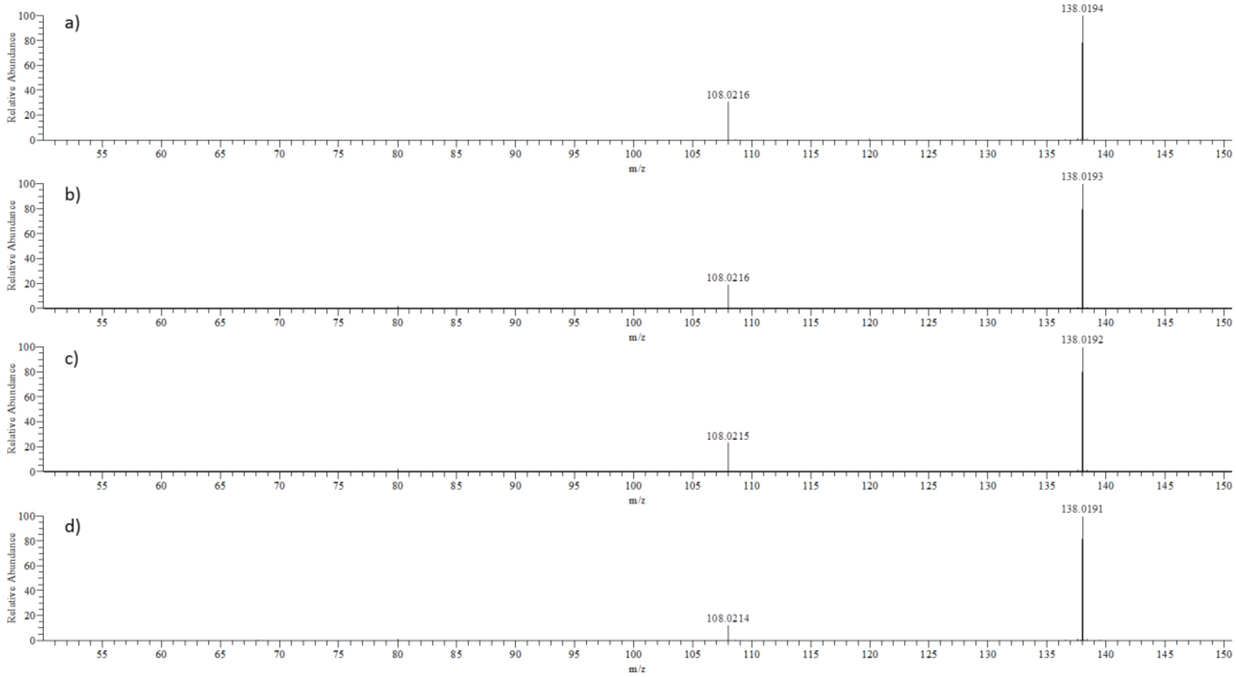

Figure S2. MS^2 spectra for precursor ions of mono-nitrotoluenes, m/z 138.0197 [M-H]$^-$, and its fragment m/z 108.0217 [M-NO]$^{\bullet -}$ a) 2-nitrophenol in ESI-; b) 3-nitrophenol in ESI-; c) 3-nitrophenol in APCI-; d) 4-nitrophenol in APCI-.

7.13E+04	1.94E+05	4.07E+05	5.05E+05	5.70E+05	6.98E+05	7.83E+05	8.84E+05	9.41E+05	1.09E+06	1.20E+06	1.33E+06	1.66E+06	1.97E+06	2.22E+06	2.14E+06	8.71E+06	1.17E+07	Intensity
0.999	1.000	1.000	1.000	0.614	0.641	0.960	0.068	0.132	8.01E-05	1.37E-05	1.00E-05	1.93E-12	4.32E-13	4.21E-13	4.21E-13	4.21E-13	4.21E-13	6.33E+04
	0.990	0.958	0.691	0.040	0.045	0.262	0.001	0.003	3.06E-07	4.46E-08	3.19E-08	4.24E-13	4.21E-13	4.21E-13	4.21E-13	4.21E-13	4.21E-13	7.13E+04
		1.000	1.000	0.788	0.810	0.991	0.133	0.238	0.000	4.13E-05	3.05E-05	5.74E-12	4.62E-13	4.20E-13	4.20E-13	4.21E-13	4.21E-13	1.94E+05
			1.000	0.906	0.919	0.999	0.230	0.377	0.001	0.000	8.32E-05	1.74E-11	5.53E-13	4.21E-13	4.21E-13	4.21E-13	4.21E-13	4.07E+05
				0.997	0.998	1.000	0.597	0.773	0.005	0.001	0.001	2.63E-10	2.49E-12	4.22E-13	4.22E-13	4.21E-13	4.21E-13	5.05E+05
					1.000	1.000	1.000	1.000	0.256	0.096	0.080	1.77E-07	1.62E-09	2.00E-12	2.00E-12	4.21E-13	4.21E-13	5.70E+05
						1.000	1.000	1.000	0.236	0.087	0.072	1.48E-07	1.35E-09	1.73E-12	1.73E-12	4.21E-13	4.21E-13	6.98E+05
							0.983	0.997	0.093	0.030	0.024	5.45E-08	6.01E-10	1.21E-12	1.21E-12	4.21E-13	4.21E-13	7.83E+05
								1.000	0.924	0.707	0.657	2.23E-05	2.64E-07	3.05E-10	3.05E-10	4.21E-13	4.21E-13	8.84E+05
									0.808	0.523	0.471	7.40E-06	8.15E-08	8.95E-11	8.95E-11	4.21E-13	4.21E-13	9.41E+05
										1.000	1.000	0.028	0.001	2.11E-06	2.11E-06	4.21E-13	4.21E-13	1.09E+06
											1.000	0.092	0.004	1.32E-05	1.32E-05	4.21E-13	4.21E-13	1.20E+06
												0.111	0.005	1.79E-05	1.79E-05	4.21E-13	4.21E-13	1.33E+06
													1.000	0.620	0.620	4.21E-13	4.21E-13	1.66E+06
														0.995	0.995	4.21E-13	4.21E-13	1.97E+06
															1.000	4.21E-13	4.21E-13	2.22E+06
																4.21E-13	4.21E-13	2.14E+06
																	4.21E-13	8.71E+06

Figure S3. Measure of significance (p-values) for FR_{50} (Fig. S.4) using one-factor ANOVA (α = 0.05) based on intensity. Highlighted values are statistically significant at 95% confidence level (green), 90% confidence level (yellow) for 4-ClBP).

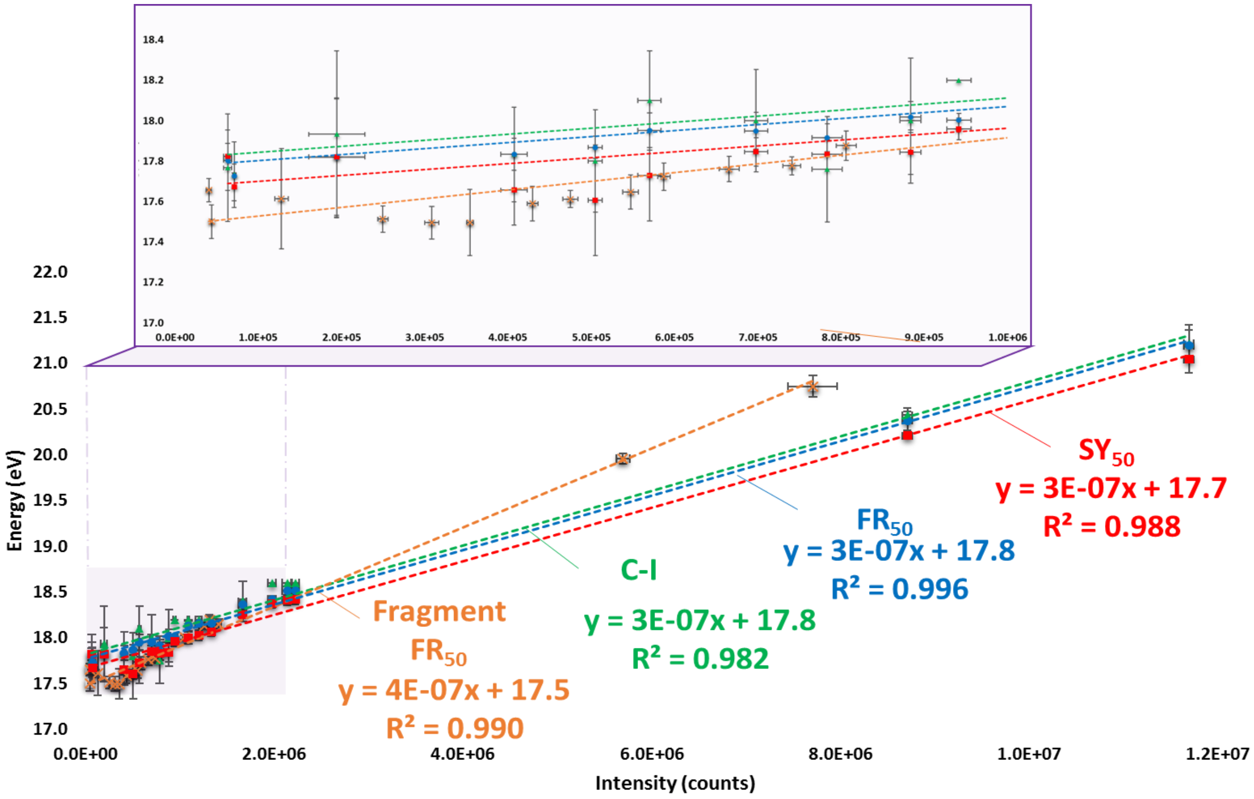

Figure S4. 4-ClBP's FR_{50}, SY_{50} and C-I dependence on concentration (expressed as intensity) from Table 1. Above inset shows expansion of region 0 to 1.0E06 intensity. FR_{50}, SY_{50} and C-I have a linear dependency with respect to ion intensity over the entire tested intensity range for 4-ClBP, with R^2 > 0.98 over three orders of magnitude.

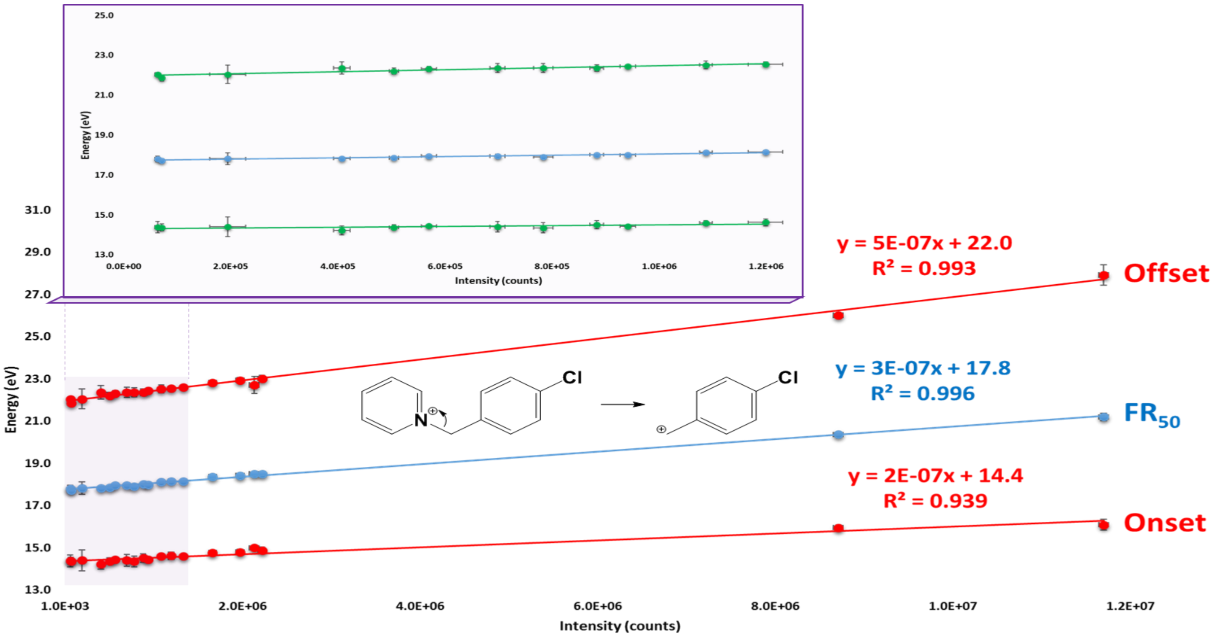

Figure S5. Dependence of Onset, FR_{50} and Offset parameters on concentration (expressed as intensity) for Fragmentation Resilience method for 4-CIBP.

Table S1. Statistical significance (p-values at α=0.05) between breakdown energies of FR_{50}, SY_{50} & C-I methods (Fig. S.4 & Table 1), various intensities using ANOVA single factor model with post-hoc Tukey HSD work up; statistical significance between FR_{50} values of precursor & fragment ion for FR method only. Highlighted values are statistically significant at 95% confidence level.

NI (counts)	p-values			
	m-SY_{50} = C-I	FR_{50} = C-I	FR_{50} = *m*-SY_{50}	FR_{50} = Frag. FR_{50}
6.33E+04	1.000	1.000	1.000	0.829
7.13E+04	1.000	1.000	1.000	0.050
1.94E+05	1.000	1.000	1.000	0.145
4.07E+05	0.999	1.000	0.999	5.6E-05
5.05E+05	0.995	1.000	0.683	5.4E-07
5.70E+05	0.030	1.000	0.949	1.6E-10
6.98E+05	1.000	1.000	1.000	1.9E-06
7.83E+05	1.000	1.000	1.000	0.002
8.84E+05	1.000	1.000	1.000	6.9E-07
9.41E+05	0.860	0.993	1.000	0.002
1.09E+06	0.987	1.000	1.000	6.2E-07
1.20E+06	0.999	1.000	1.000	2.5E-07
1.33E+06	1.000	1.000	1.000	0.001
1.66E+06	1.000	1.000	1.000	1.8E-06
1.97E+06	0.934	1.000	1.000	0.000
2.22E+06	0.999	1.000	1.000	2.0E-06
2.14E+06	0.997	1.000	1.000	9.3E-05
8.71E+06	0.946	1.000	1.000	1.1E-14
1.17E+07	1.000	1.000	1.000	1.8E-06

Table S2. Dependence of FREMS values on source parameters, tested on Thermo Scientific LTQ-Orbitrap XL™ using 4-ClBP. To ascertain what effects front-end conditions on breakdown energies, all accessible conditions were tested

Parameter	Day	NI (counts)	C-I (eV)	*m*-SY_{50} (eV)	FR_{50} (eV) m/z 204.0575	FR_{50} (eV) m/z 125.0153
STM*	368	1.22E+06	17.8 ± 0.2[a] (17.6)[b]	17.5 ± 0.1 (17.4)	17.3 ± 0.1 (17.3)	17.0 ± 0.1 (17.0)
0 eV in-source CID	368	1.18E+06	17.7 ± 0.1 (17.6)	17.6 ± 0.1 (17.4)	17.4 ± 0.1 (17.4)	17.1 ± 0.1 (17.1)
35 eV in-source CID	368	1.38E+05	16.6 ± 0.1 (16.6)	16.5 ± 0.1 (16.4)	16.5 ± 0.3 (16.4)	16.1 ± 0.2 (16.1)
STD Method	370	1.27E+06	17.6 ± 0.0 (17.6)	17.5 ± 0.1 (17.3)	17.3 ± 0.0 (17.3)	17.0 ± 0.1 (17.0)
35Sheath15Aux0Sweep	370	9.61E+05	17.2 ± 0.2 (17.0)	17.2 ± 0.1 (17.0)	17.0 ± 0.1 (17.0)	16.7 ± 0.1 (16.7)
35Sheath15Aux2Sweep	370	1.91E+05	16.5 ± 0.1 (16.4)	16.5 ± 0.1 (16.3)	16.2 ± 0.1 (16.2)	15.9 ± 0.2 (15.9)
15Sheath3Aux2Sweep	370	2.44E+05	16.4 ± 0.3 (16.2)	16.4 ± 0.1 (16.2)	16.2 ± 0.1 (16.2)	16.0 ± 0.1 (16.0)
3.5 kV Spray	370	2.53E+05	16.6 ± 0.2 (16.4)	16.5 ± 0.1 (16.3)	16.3 ± 0.1 (16.3)	16.0 ± 0.1 (16.0)
STD Method	371	1.03E+06	17.3 ± 0.3 (17.0)	17.3 ± 0.1 (17.0)	17.1 ± 0.1 (17.1)	16.7 ± 0.1 (16.7)
Ion Max #2	371	8.27E+05	16.9 ± 0.3 (16.6)	16.9 ± 0.1 (16.7)	16.6 ± 0.1 (16.6)	16.4 ± 0.1 (16.4)
Ion Max #1	371	1.31E+06	17.4 ± 0.3 (17.2)	17.2 ± 0.2 (17.2)	17.0 ± 0.0 (17.0)	16.8 ± 0.0 (16.8)
Ion Optics Variables All	371	2.81E+05	16.3 ± 0.3 (16.2)	16.3 ± 0.1 (16.2)	16.2 ± 0.1 (16.2)	15.9 ± 0.1 (15.8)
STM*	372	1.32E+06	17.4 ± 0.2 (17.2)	17.3 ± 0.1 (17.2)	17.1 ± 0.1 (17.1)	16.8 ± 0.1 (16.8)

*STM – no in-source CID, 15Sheath3Aux0Sweep gas flows, 4.5 kV ionization voltage, Ion Max #1 housing (with #1 probe).

Automatic Gain Control (AGC) Parameters Dependence. Ion Population Injection parameter allows the user to set the number of ions entering the ion trap (IT) or Orbitrap (FT), i.e. Full MS, SIM or MS^n. Low values may result in shorter injection time; too many ions may affect isolation/fragmentation efficiency and mass accuracy. All IT and FT ion population parameters, with the exception of FT-MS^n, had no effect on FREMS values (p-value > 0.05, n = 38) testing at each extreme level (Tables S.3, S.4). This was not surprising as FREMS MS^2 detection takes place in the Orbitrap. No significant mass accuracy defects were observed for varying amount of ions (1E3 to 1 E6 < 2 ppm; 1E7 ~ 6ppm). Despite isolation and fragmentation taking place inside the ion trap, no IT parameter had any effect on FREMS values. The deviation of FREMS values to lower values started once the number of ions fell below 1000 (Fig.S.6,17.4%; p-value< 0.05). Lower injection amounts resulted in shorter run times, but much noisier signal. No signal was observed below 100 injected ions. No statistical significance was observed between all FT and IT parameters set to maximum (1E7 and 1E6, respectively) and a 1-point calibration (STM) for that day. The cross-intersect method was statistically significant in some cases when compared to m-SY (p-value < 0.05 for n=14 of 109 tested pairs), while m-SY and FR had no differentiation. To avoid artificial energy shifts, operation in ion range 5E4-1E7 is advised.

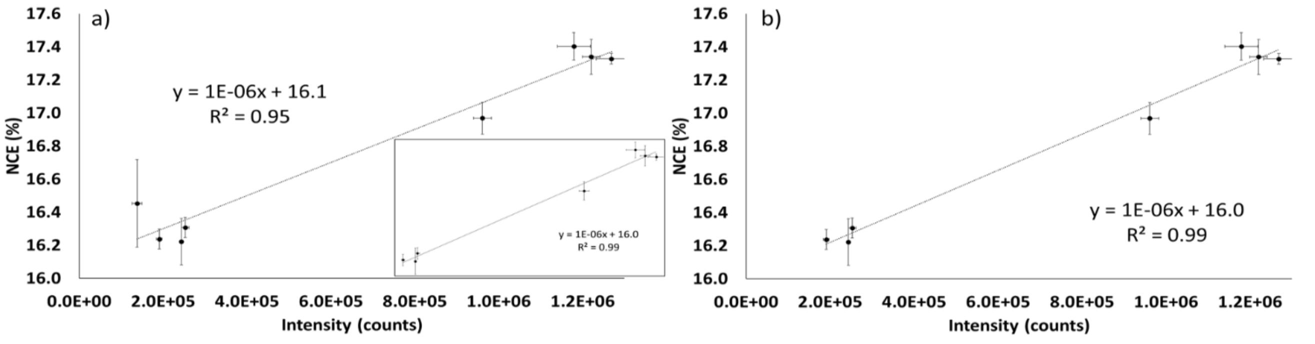

Figure S6. FR_{50} dependence on a) gas flows & in-source CID; b) ion optics & ionization source housing.

Table S3. Effects of Orbitrap's Automatic Gain Control Ion Population parameters on averaged FREMS values using 4-ClBP on Thermo Scientific LTQ-Orbitrap XL™.

		FREMS			FT Ion Population Injection			
Day	NI (counts)	C-I (eV)	*m*-SY_{50} (eV)	FR_{50} (eV)	FT Full MS	FT SIM	FT MSn	Notes
3*	1.11E6	18.2	18.0	18.1	2.00E5	1.00E5	1.00E5	*STM
3*	1.26E6	18.2	18.0	18.2	2.00E5	1.00E5	1.00E5	*STM
3*	1.36E6	18.2	18.0	18.2	2.00E5	1.00E5	1.00E5	*STM
10	2.30E6	18.4	18.2	18.2	1.00E7	1.00E5	1.00E7	
10	1.42E6	18.2	18.0	18.1	1.00E6	1.00E5	1.00E7	
10	1.32E6	18.2	18.0	18.1	1.00E5	1.00E5	1.00E7	
12	1.43E6	18.2	17.9	18.1	1.00E5	1.00E5	1.00E7	1
12	1.53E6	18.2	17.8	18.0	1.00E4	1.00E5	1.00E7	
12	1.52E6	18.2	17.9	18.0	1.00E0	1.00E5	1.00E7	
12	1.29E6	18.2	17.8	17.9	1.00E7	1.00E5	1.00E6	(0)
12	1.33E6	18.2	17.9	18.1	1.00E7	1.00E5	1.00E5	
12	1.05E6	18.2	17.9	18.0	1.00E7	1.00E5	1.00E4	
12	3.55E6	17.6	17.7	17.9	1.00E7	1.00E5	1.00E3	2; FA (7)
16	1.98E6	17.2	17.2	17.4	1.00E7	1.00E5	1.00E3	2, 3(<1E3); LV
16	1.00E6	17.6	17.6	17.7	1.00E7	1.00E0	1.00E7	(-); LV
16	1.06E6	17.6	17.6	17.7	1.00E7	1.00E7	1.00E7	LV
17	1.05E6	18.2	18.0	18.0	1.00E7	1.00E7	1.00E7	1

Day – days since start of experiment; *Values are taken from calibration Table 1 for comparison under standard conditions; () accurate mass shift (-) below, (+) above or at exact mass (0); [1]rerun of sample under identical conditions from sample above it; [2]noisy data (high background noise, but might be mechanical issue, e.g. stalling syringe pump or air bubbles in solution = no signal); [3]no signal under this conditions (below intensity threshold); LV – lower C-I, SY_{50} and FR_{50} values across all measurements on this date (some uncontrolled parameter(s) affecting results); FA – faster acquisition (# of curves); *STM – 1-point calibration.

Table S4. Effects of linear ion trap's Automatic Gain Control Ion Population parameters on averaged FREMS values using 4-ClBP on Thermo Scientific LTQ-Orbitrap XL™.

		FREMS			IT Ion Population Injection				
Day	NI (counts)	C-I (eV)	*m*-SY_{50} (eV)	FR_{50} (eV)	IT Full MS	IT SIM	IT MSn	IT Zoom	Notes
3*	1.11E6	18.2	18.0	18.1	3.00E4	1.00E4	1.00E4	3.00E3	*STM
17	1.20E6	18.2	18.0	18.0	1.00E0	1.00E4	1.00E4	3.00E3	
17	1.16E6	18.2	18.0	18.2	1.00E6	1.00E4	1.00E4	3.00E3	
18	1.24E6	18.2	18.0	18.2	1.00E6	1.00E4	1.00E4	3.00E3	1,‡(10)
18	1.26E6	18.2	18.0	18.2	3.00E4	1.00E0	1.00E4	3.00E3	
19	1.43E6	18.2	17.9	18.0	3.00E4	1.00E0	1.00E4	3.00E3	1
19	1.34E6	18.2	17.9	17.9	3.00E4	1.00E6	1.00E4	3.00E3	
19	1.30E6	18.2	17.8	17.9	3.00E4	1.00E4	1.00E0	3.00E3	C
19	1.30E6	18.2	17.8	17.9	3.00E4	1.00E4	1.00E6	3.00E3	
19	1.00E6	17.6	17.7	17.9	3.00E4	1.00E4	1.00E6	3.00E3	1; NS
19	1.04E6	18.2	17.8	17.9	3.00E4	1.00E4	1.00E4	1.00E0	
22	1.16E6	17.6	17.4	17.6	3.00E4	1.00E4	1.00E4	3.00E3	*STM
22	1.29E6	17.6	17.5	17.7	3.00E4	1.00E4	1.00E4	1.00E0	1
22	1.30E6	17.6	17.5	17.6	3.00E4	1.00E4	1.00E4	1.00E6	T_r-2
22	1.35E6	17.6	17.5	17.6	1.00E6	1.00E6	1.00E6	1.00E6	All FT=1E7

Day – days since experiment start; *Values from calibration Table 1 for comparison under standard conditions; [1]rerun of sample under identical conditions as above; ‡ - instrument has not been calibrated (#days); C- calibrated prior to this run; NS – freshly prepared standard at same concentration; T_r±X = average room temperature (73F) ± degrees (F); *STM – 1-point calibration

***Maximum* Inject Time.** This AGC variable affected FR_{50}, SY_{50} and C-I values only in FT-MS^n and not in other modes (Tables S.5-S.7). It controls time duration for ion batch accumulation before send off to the detector and competes with the *Ion Population Injection* parameter. When *Maximum Inject Time* was set to 1 ms, it sped up data acquisition, producing an extra breakdown curve for the same time interval as the standard method (100 ms). However, as this reduced the amount of ion sent to MS for analysis, it lowered the S/N ratio and diminished efficiency of statistical modeling. In contrast, acquisition times got longer as injection time was increased; only 2 breakdown curves were collected in 1000 ms. There was a linear upward trend for FREMS values with respect to the *Inject Time* parameter (Fig. S.7a), attributed to more efficient collisional cooling from longer residence times. As internal energy gets dampened during vibrational relaxation, it would require more energy to initiate fragmentation. Beyond 1000 ms time interval, the *Ion Population* parameter began to compete with *Inject Time*, making it unreliable and breaking a linear trend. *Maximum Inject Time* had no influence on total ion current over the tested range, and therefore had its own direct effect on FR_{50}, m-SY_{50} and C-I values (i.e. it cannot be intensity-adjusted). This parameter should be fixed (e.g. 10 ms) or calibrated over a linear range before using FREMS for compound differentiation. No statistical significance among FREMS values (p-value > 0.05) was observed for n = 46 pairs.

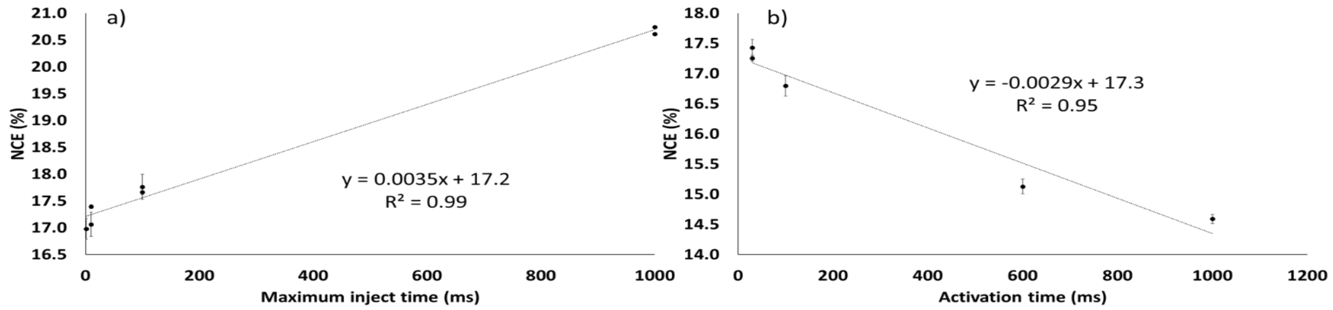

Figure S7. FR_{50} relative to a) Maximum Inject Time b) Activation Time, in FT-MS^n.

Table S5. Effects of Inject Times (ms) in Orbitrap analyzer on FREMS values using 4-ClBP on Thermo Scientific LTQ-Orbitrap XL™.

		FREMS			FT Inject Time			
Day	NI (counts)	C-I (eV)	*m*-SY_{50} (eV)	FR_{50} (eV)	FT Full MS	FT SIM	FT MSn	Notes
22	1.24E6	17.6	17.5	17.7	10	50	100	*STM
22	2.61E6	17.2	17.3	17.0	10	50	1	2; FA (7)
22	1.61E6	17.2	17.0	17.1	10	50	10	2; FA (7)
23	1.34E6	17.6	17.4	17.4	10	50	10	1; 2; FA (7)
23	8.04E5	20.4	20.5	20.6	10	50	1000	2; SA (2)
23	8.52E5	21.0	20.8	20.7	10	50	1000	1
23	9.77E5	22.8	22.7	22.8	10	50	8000	2; SA (1)
23	1.33E6	17.6	17.6	17.8	10	50	100	*STM
24	1.25E6	17.6	17.8	17.9	10	50	100	*STM; 1; NS
24	1.19E6	17.6	17.7	17.8	1	50	100	
24	1.06E6	17.6	17.7	17.8	8000	50	100	
24	1.01E6	17.6	17.6	17.8	10	1	100	
24	9.86e5	17.6	17.6	17.7	10	8000	100	

Day – days since initiation of experiment; [1]rerun of the sample under the identical conditions as sample above it; [2]noisy data (usually high background noise, but might be mechanical issue, e.g. stalling syringe pump or air bubbles = no signal); FA–faster acquisition (# of curves); SA–slower acquisition (# of curves); *STM–standard method – 1-point calibration.

Table S6. Effects of Inject Times (ms) in IT analyzer parameters on averaged FREMS values using 4-ClBP on Thermo Scientific LTQ-Orbitrap XL™.

		FREMS			IT Inject Time				
Day	NI (counts)	C-I (eV)	*m*-SY_{50} (eV)	FR_{50} (eV)	IT Full MS	IT SIM	IT MSn	IT Zoom	Notes
26	8.82E5	17.6	17.5	17.6	10	50	100	50	*STM
26	8.11E5	17.6	17.3	17.5	1	50	100	50	
26	8.03E5	17.4	17.3	17.4	8000	50	100	50	
26	8.70E5	17.6	17.3	17.4	10	1	100	50	
26	8.85E5	17.6	17.4	17.5	10	8000	100	50	
26	8.54E5	17.6	17.4	17.5	10	50	1	50	
26	8.74E5	17.6	17.4	17.5	10	50	8000	50	
26	8.72E5	17.6	17.4	17.5	10	50	100	1	
26	8.77E5	17.6	17.5	17.6	10	50	100	8000	

Day – amount of time passed after initiation of experiment; *STM – standard method – 1-point calibration.

Activation Time Dependence. Activation Time parameter allows the user to set how long RF is used for fragmentation and has a direct link to studies of kinetic shift.[3,5,6] It is different from Inject Time, but has some similar attributes. Increasing Activation Time also increases the acquisition run; two curves were obtained in 1000 ms instead of six for the standard method (30 ms), but in contrast to Inject Time, an increase in Activation Time produced a downward trend for FREMS values (R^2 = 0.95, Fig. S.7b), indicating shorter RF pulses induces lower ion kinetic energy and less fragmentation. Because the correlation of Activation Time with FREMS values was not perfectly linear, this parameter was fixed at 30 ms. For most observations there was no statistical significance among FR, m-SY and C-I methods (p-value > 0.05 for n = 16 pair observations, exception 600 ms two observations, Fig. S.7b).

Wideband Activation and Activation Q Parameters Dependence – Fixed Values. Wideband Activation is a parameter that provides RF voltage with a wide range of excitation frequencies. While it might be useful for multiple fragmentation of the precursor and its fragment ions, this parameter can only be switched on or off without control for energy deposition. FERMS techniques used with Wideband Activation were statistically significant (p-value < 0.05 for all observations) in respect to the standard method with FR_{50} at intensity 2.5E6 (22.4 ± 0.3 eV and 18.7 ± 0.3 eV, respectively). Because this parameter offers no advantages for FREMS methods, it should be avoided. Another parameter that directly affects FREMS values was Activation Q, which allows adjusting of the RF frequency used in fragmentation. Typically, a smaller value for this parameter results in less energy deposition/fragmentation but allows for observation at lower m/z fragments, extending the ion trap so-called “1/3 rule.”[42] Experiments lowering Activation Q to 0.05 produced no observable fragment, m/z 125.0153. With less energy deposition, greater FREMS values were expected with comparison to the standard (Q = 0.25) method; instead, the opposite was observed with FR_{50} of 12.4 ± 0.2 eV (3.5E5 intensity) vs. 17.5 ± 0.3 eV (7.4E5 intensity), respectively. Intensity-adjusted values were still statistically significant (p-value < 0.05) for this observation. Because this parameter affects multiple variables, i.e,. FREMS values and fragment presence/absence, it is advised to fix this value for all observations (e.g. 0.25 as in STM).

Other Parameters – No Dependence. The Microscans (μs) parameter affects how many scans are summed in the background to produce a final, single output. Only FT-MS^n mode had any influence on the data output – increasing the number of Microscans increases the acquisition time. The relationship was almost linear as it produced 17 breakdown curves for 1 μs, 3 curves for 6 μs and 1 curve for 15 μs, while no FREMS values were affected (p-value > 0.05, Tables S.8, S.9). A single breakdown curve for 15 μs had low signal-to-noise, resulting in high fitting error for the statistical model and was omitted from consideration for FR_{50} comparison; otherwise, no differences between FREMS methods were observed (p-value > 0.05 for n = 36 pair observations). Because Microscans were the most influential parameter in reducing acquisition times without affecting FREMS values, it is advised to set the value to 1 μs and, if necessary, perform any smoothing post data acquisition. Remainder of controllable parameters: Mass Range, Data Type, Resolution had no effect on FREMS values (p-values > 0.05 for all, Table S.10). However, lower resolution sped up acquisition time, since it lowered transient times inside the Orbitrap.

Table S7. Dependence of FREMS values on Activation Time (ms) on Thermo Scientific LTQ-Orbitrap XLTM, 4-ClBP.

**Parameter**	**Day**	**NI (counts)**	**C-I (eV)**	***m*-SY_{50} (eV)**	**FR_{50} (eV) m/z 204.0575**	**FR_{50} (eV) m/z 125.0153**
*STM	366	2.63E+06	17.8 ± 0.3[a] (17.6)[b]	17.7 ± 0.2 (17.5)	17.4 ± 0.1 (17.4)	17.2 ± 0.2 (17.2)
1000 ms	366	1.99E+06	15.3 ± 0.7 (14.8)	15.2 ± 0.1 (15.0)	14.6 ± 0.1 (14.6)	14.3 ± 0.0 (14.3)
600 ms	366	2.10E+06	16.1 ± 0.3 (15.8)	15.8 ± 0.1 (15.7)	15.1 ± 0.1 (15.1)	15.3 ± 0.1 (15.3)
100 ms	366	2.20E+06	17.2 ± 0.3 (17.0)	17.2 ± 0.1 (17.0)	16.8 ± 0.2 (16.8)	16.9 ± 0.1 (16.9)
10 ms	366	2.30E+06	21.5 ± 0.2 (21.4)	21.5 ± 0.1 (21.3)	21.6 ± 0.2 (21.6)	20.5 ± 0.1 (20.5)
*STM	366	2.35E+06	17.7 ± 0.1 (17.6)	17.5 ± 0.1 (17.2)	17.3 ± 0.1 (17.3)	17.0 ± 0.1 (17.0)

Day – days since initiation of experiment; NI – Normalized intensity; C-I – cross-intersect method; SY_{50} – inflection point of *m*-SY curve; FR_{50} – inflection point of FR curve; [a] standard deviation based on n=6 replicates; [b] statistical analysis of all combined curves (single value); *STM – standard method – 1-point calibration .

Table S8. Effects of number of Microscans in FT analyzer on averaged FREMS values using 4-ClBP on Thermo Scientific LTQ-Orbitrap XL™.

		FREMS			FT Microscans			
Day	NI (counts)	C-I (eV)	*m*-SY_{50} (eV)	FR_{50} (eV)	FT Full MS	FT SIM	FT MSn	Notes
22*	1.16E6	17.6	17.4	17.6	2	1	3	*STM
22	1.17E6	17.6	17.4	17.5	1	1	3	
22	1.29E6	17.6	17.3	17.4	5000	1	3	
22	1.29E6	17.6	17.4	17.7	2	5000	3	
22	1.66E6	17.6	17.5	17.6	2	1	1	FA (17)
22	1.24E6	17.6	17.5	17.6	2	1	6	SA (3)
22	1.49E6	17.2	17.0	16.5	2	1	15	2; SA (1)

Day –days since initiation of experiment; [2]noisy data (usually high background noise, but might be mechanical issue, e.g. stalling syringe pump or air bubbles in the solution = no signal); FA – faster acquisition (# of curves); SA – slower acquisition (# of curves); *STM – standard method – 1-point calibration.

Table S9. Effects of number of Microscans in IT analyzer parameters on averaged FREMS values using 4-ClBP on Thermo Scientific LTQ-Orbitrap XL™.

		FREMS			IT Microscans				
Day	NI (counts)	C-I (eV)	*m*-SY_{50} (eV)	FR_{50} (eV)	IT Full MS	IT SIM	IT MSn	IT Zoom	Notes
24*	1.25E6	17.6	17.8	17.9	4	1	3	1	*STM; NS
24	9.71E5	17.6	17.6	17.6	1	1	3	1	
24	9.23E5	17.6	17.6	17.7	5000	1	3	1	
24	9.14E5	17.6	17.6	17.8	4	5000	3	1	
24	8.84E5	17.6	17.7	17.7	4	1	5000	1	
25	6.63E5	18.2	17.8	18.0	4	1	5000	1	1
25	7.90E5	17.6	17.7	18.0	4	1	3	5000	

Day – days since initiation of experiment; NS – freshly prepared standard at the same concentration; *STM – standard operating conditions – 1-point calibration.

Table S10. Effects of Scan Range, Resolution and Data Acquisition Type on FREMS values using 4-ClBP on Thermo Scientific LTQ-Orbitrap XL™.

		FREMS			Misc. Parameters			
Day	NI (counts)	C-I (eV)	*m*-SY_{50} (eV)	FR_{50} (eV)	Mass Range	Res	Data Type	Notes
30	5.94E5	17.6	17.7	17.9	Normal	15000	Profile	*STM
30	6.97E5	17.6	17.7	17.9	Normal	15000	Profile	*STM; NS
30	7.05E5	17.6	17.7	17.7	Normal	15000	Profile	*STM
30	6.86E5	17.6	17.7	17.7	Normal	15000	Profile	*STM
30	7.13E5	17.6	17.6	17.8	Normal	15000	Profile	*STM
31	6.76E5	17.6	17.6	17.7	Normal	15000	Profile	*STM
31	7.92E5	17.6	17.5	17.6	High	15000	Profile	
31	6.34E5	17.2	17.3	17.3	Normal	100000	Profile	2; SA (2)
31	6.95E5	17.6	17.4	17.6	Normal	15000	Centroid	
31	6.98E5	17.6	17.4	17.5	Normal	15000	Profile	*STM

Day – days since initiation of experiment; [2]noisy data (usually high background noise, but might be mechanical issue, e.g. stalling syringe pump or air bubbles in the solution = no signal); Res – resolution; SA – slower acquisition (# of curves); *STM – standard method – 1-point calibration.
